# A geographic information system-based approach of flood hazards modelling, Paschim Medinipur district, West Bengal, India

**DOI:** 10.4102/jamba.v10i1.518

**Published:** 2018-03-26

**Authors:** Kishor Dandapat, Gopal K. Panda

**Affiliations:** 1 Department of Geography, Utkal University, India

## Abstract

From the beginning of civilisation, human beings have preferred living on the river banks which have been the most vulnerable areas of flood hazards and consequent disasters. During the monsoon period, in many developing countries of south-east Asia, flood hazards and disasters have been a serious challenge for their development. Most of the rivers exceed their normal channel capacity attaining the flood stage and frequently overflow their banks, causing great havoc to the life and property of the people. Flooding is a very serious problem in many districts of West Bengal. The prime concern of delineation of flood-prone areas is to regulate the land use in the flood-prone areas to restrict damage potential and also mitigate the negative effects of floods on people and the economy. In a regulated way, flood-prone areas are required to be developed. Because, on one hand, it is to be ensured that existing hazard and flood damage potential do not increase and new developmental works become a step towards mitigation of disaster risk. In a perspective view, the demarcation and identification of flood-prone areas of different magnitudes, frequencies and return periods on a large-scale map seem to have great importance. Satellite-derived flood maps from 2007 to 2016 have been applied to form a flood frequency map and the same as a group of flood depth maps has been employed to produce the Flood Damage Map for depth data of flood. Finally, the modelling of flood hazards has been directed by envisaging amalgamation of Flood Depth and Flood Affected Frequency. Then the final flood hazard map amalgamated with population and housing data has been used to ascertain the flood disclosure for these two components. Flood hazard analysis in the study area revealed that 24% of the population has been located in high flood hazard zones, where 39% of human settlements are located in different flood hazard zones.

## Introduction

A large portion of Paschim Medinipur district in the state of West Bengal in eastern India is vulnerable to flooding. The area of the district is 9295 km^2^, out of which 1952 km^2^ is flood prone. The district has four major rivers: Kangsabati, Silabati and Subarnarekha, which have their origin from Chotanagpur and Ranchi plateaus; and Kaliaghai River, which originates in the said district and is also very much flood prone in rainy season. All these rivers are commanding huge catchment areas in and outside the district and bring enormous volumes of water during the rainy season. The frequent floods occurring in great magnitude have left their indelible marks over this region since its history and their ravages tell a grim and sorrowful tale of the suffering of the people. The annual rainfall of the district varies from 1400 mm to 1500 mm, while the annual temperature ranges from 9 °C to 43 °C. The maximum rainfall occurs (75%) during the monsoon periods, from May to September. Heavy rainfall in Chotonagpur plateau results in large inflow into the reservoirs of Chandil, Galudi and Durgapur and heavy rain in Bankura and adjacent districts results in large inflow into the reservoir of Kangsabati. All these reservoirs have failed to control the flood discharge into the rivers in the later part of the rainy season to control the heavy flows. The heavy discharge within a short span of time with onrush of water through the rivers causes inundation, submergence and water logging in vast areas. Very often river banks collapse with high floods and create havoc to the life and property of the people in low lying regions of the district. The high flood events have been determined on the basis of the river gauge site and which flood year crossed the danger level that is addressed as a high flood event. The recurrence interval or return period *T* in years is calculated by using the Weibull’s formula: *T* = *N*+1/*M*, where *N* is the total number of events and *M* is the magnitude of high flood events (see Table 1).

**TABLE 1:** High flood frequencies and their recurrence interval.

River	Total time span	Number of high flood events	Recurrence interval of high magnitude flood events
Kangsabati	1950–2015 = 65 years	31 (high magnitude floods)	65+1/31 = 2.13 (one each in 2.13) years)
Silabati	1978–2016 = 38 years	10 (high magnitude floods)	38+1/10 = 3.9 (one each in 3.9 years)
Kaliaghai	2001–2016= 15 years	14 (high magnitude floods)	15+1/14 = 1.14 (one each in 1.14) years)
Subarnarekha	2005–2016 = 11 years	3 (high magnitude floods)	11+1/3 = 4 (one each in 4 years)

**FIGURE 1:**
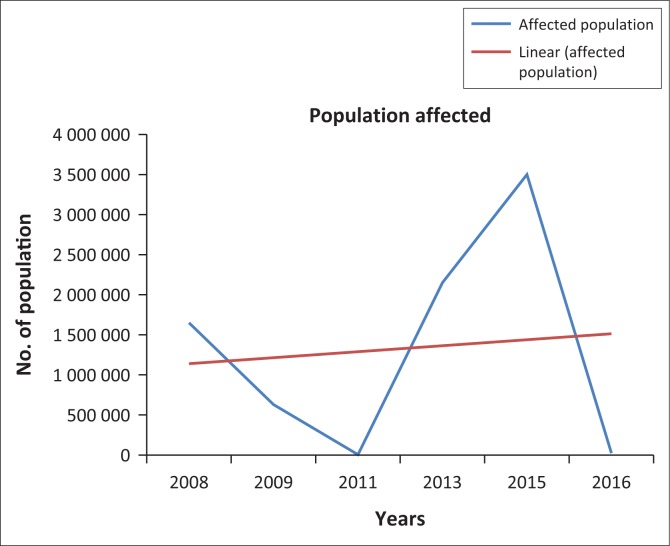
Affected population during some selected flood years.

**FIGURE 2:**
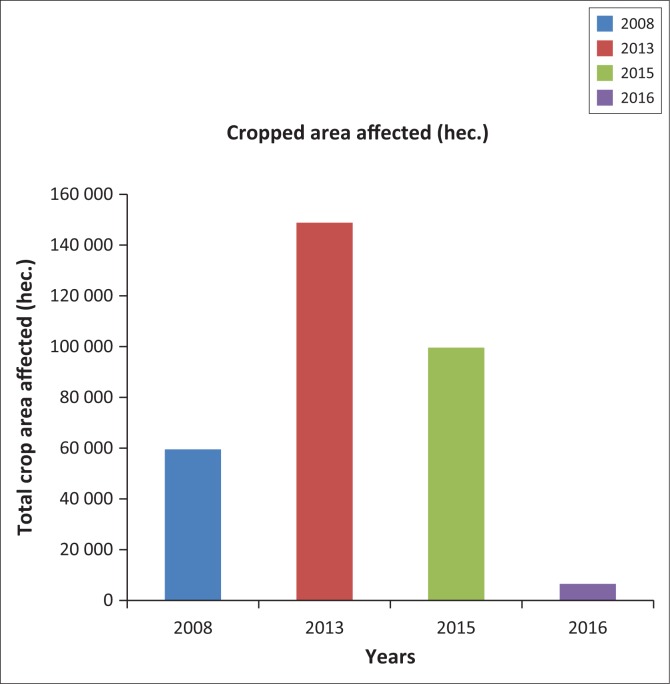
Crop area affected during some selected flood years.

The damage caused by floods during the period 2008–2016 revealed that maximum damage occurred in the year 2008, 2009, 2013 and 2015. The damage caused by floods in these 4 years brought immense misery to the people of the district. The population most affected by heavy rain and flood water discharge from different dams was in 2015 and it has been estimated to be 35 lakhs (see Figure 1) as per report of the Disaster Management Department, which supersedes all other damages recorded earlier. In 2015, the flood damage occurred over 3200 km^2^ of area and 90 374 ha of crop area (see Figure 2) were damaged. It has been estimated that 56 214 houses were fully and 211 411 houses were partly damaged by the 2015 flood. It was also estimated that 11 267 villages were affected and 13 persons died besides a large number of cattle population. Thus, the damages occur in several forms. The agricultural lands with standing paddy plants remain completely submerged for several days in low lying areas. Human habitation and crops are washed away by strong currents, causing numerous breaches across the roads and embankments. Communication links get dislocated and damaged. The researcher extensively visited different places of the district to have a picture of the catastrophic flood of 2015. Some people who had experiences to witness several floods in the district liked to opine that such floods had not occurred in the district during the last two decades. So, in the present context it is an important matter to identify the flood hazard zones and precautionary measures should be taken in the high flood hazard zones.

## Literature review

Many scholars and geographers have studied flood inundations and tried to determine the flood areas in different parts of the world with different approaches. In the beginning of the 19th century, attempts were made to draw inundations maps in different parts of the world. The flood hazard mapping and zoning techniques in the United States owe their origin and development to the pioneering works of several researchers including Ellis (1969), Wolman (1971) and Dingman (1975). Islam, Bala and Haque (2010) prepared a flood inundation map for Bangladesh by using moderate-resolution imaging spectroradiometer (MODIS) images of different times for their study. They also compared RADARSAT images with satellite-based remote sensing data set in their study and found a very strong correlation to derive an inundation map with a determinant coefficient of *R*^2^ = 0.96. Huang, Chen and Wu (2014) studied on dynamics of flood inundation in Murray–Darling river basin in Australia. For this study, they used time series flood data and MOIDS imagery. They used peak flood data for choosing exact MOIDS imagery to identify the submerged area of flood. Using hydrological and remotely sensed inundation data, they produced a spatiotemporal inundation map, which will be very helpful to study the eco-hydrological characteristics of the Murray–Darling river basin. Another study on flood hazard assessment was conducted by Islam and Sado (2000). In their study, the authors used National Oceanic and Atmospheric Administration advanced very high radiometric resolution (NOAA AVHRR) data with geographic information system(GIS). Different time series flood images have been used to produce flood hazard maps. One flood frequency map and a flood water depth map have been prepared as final output of flood hazard maps. Bates et al. (2006) studied the dynamics of floodplain inundation. For their study, they used airborne synthetic aperture radar (SAR) imagery. Kopili River Basin of Brahmaputra River situated in the north eastern part of India is one of the most vulnerable flood-affected river basins in India. In the last two decades, 183 flood events have been identified there. In 1977, 1988 and 1998–2015, a total of 3.89 lakh hectares land area was inundated during the time of flood. For flood hazard zonation, Sharma et al. (2016) selected a village as a unit and classified low, medium and high flood hazard zones on the basis of how many villages have been affected in a particular flood. From the study, it has been revealed that 742 villages belong to low hazard zones, 396 villages belong to medium flood hazard zones and 150 villages fall into the high zones of flood hazards.

## Objectives

India is one of the most flood-prone countries in the world. The need for flood inundation mapping as well as flood plain zoning is most essential for disaster preparedness and mitigation of floods. It is, however, to be noted that the pace of research in this area of vital human concern is rather scarce in the country. Paschim Medinipur district is also affected by flood in every year, yet no attempt has so far been made to study or map the flood intensifying zones of the district. Therefore, the prime objective of this study was to delineate a flood hazard map of the district, which would be very helpful for the local people to understand the hazards comprehensively.

## Methodology

**FIGURE 3:**
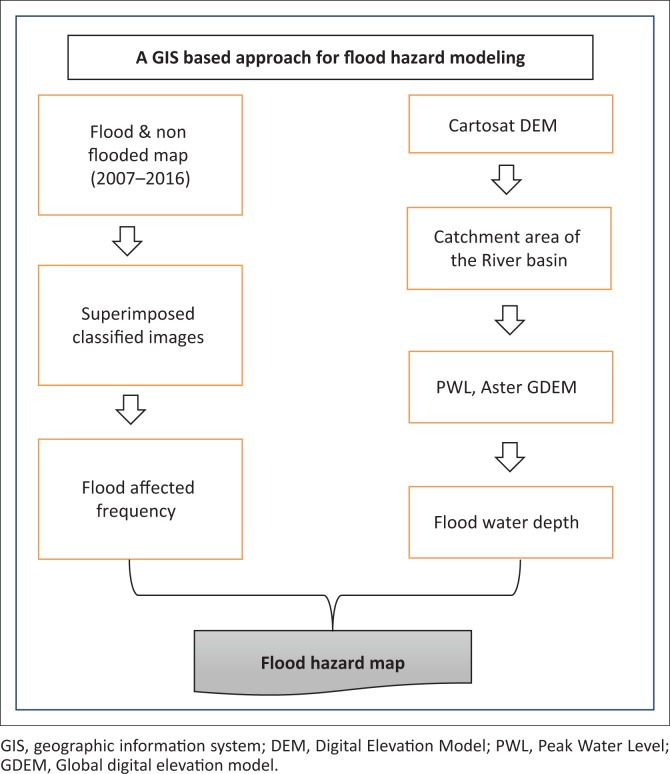
A geographic information system-based computational protocol for flood hazard mapping.

The adopted methodology of this study is shown in Figure 3, which shows the flowchart for the development of a flood hazard map. The various steps are involved in the following manner. Firstly, the flood inundation map from 2007 to 2016 has been collected from the National Remote Sensing Centre, Bhuvan, a geo-platform of the Indian Space Research Organisation. River Peak Water Level (PWL) data have been collected from the Irrigation and Waterways Department of West Bengal. Secondly, for demarcation of the river basin area, Digital Elevation Model (DEM) was derived from Bhuvan. Thirdly, to determine the flood-affected frequency map, flood inundation maps from 2007 to 2016 have been used (see the ‘Flood frequency analysis’ section) and to determine the flood water depth map, DEM and PWL of the major rivers have been used (see the ‘Flood water depth analysis’section). Finally, the flood hazard map has been developed by considering both the flood-affected frequency map and flood water depth map (see the ‘Flood hazard map preparation’ section).

## Description of the study area

**FIGURE 4:**
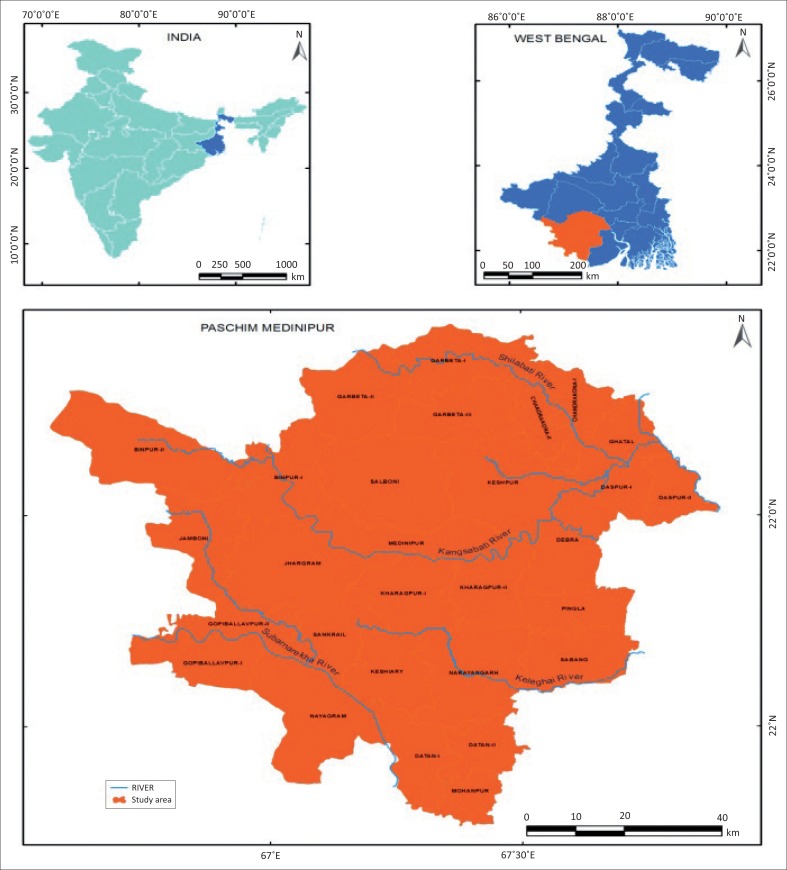
Geographical Location of the study area.

Paschim Medinipur district, located in the southern part of West Bengal, has been curved from the erstwhile Medinipur district, the then largest district of India, and came into existence in the present from 01 January 2002. It is situated between 22°57’10” north and 21°36’35” north latitude and 88°12’40” east and 86°35’55” east longitude (see Figure 4). It is bounded by Bankura district at the northern side and Purba Medinipur district at the south-eastern side. The southern boundary of the district is merged with Balasore and Mayurbhanj districts of Odisha and the western boundary is merged with Singhbhum district of Jharkhand. The geographical area of the district is 9295km^2^. The district is divided into 4 subdivisions, 29 blocks and 8 municipalities. The major rivers of the district, namely, Kangsabati, Silabati and Subarnarekha, originate from the Chotanagpur plateau on the north-west side of the district. The river Kaliaghai, another important river of the district, originates in the district itself. All the rivers flow towards south-east direction according to the variation of the topographical characteristics. The topographical slope of the district is from north-west to south-east. In the extreme south-east of the district, altitude varies from 3 m to 5 m (see Figure 4d).

## Discussion and analysis

### Flood frequency analysis

Different hydrological variables, such as flood frequency, depth of flooding, rate of water level rises, water velocity, topographical exposure and sediment load, influence flood hazards of a particular area. Two hydraulic components, that is, flood frequency and flood water depth, have been taken into consideration for the assessment and confirmation of the vulnerability of the district to flood hazards.

**FIGURE 5:**
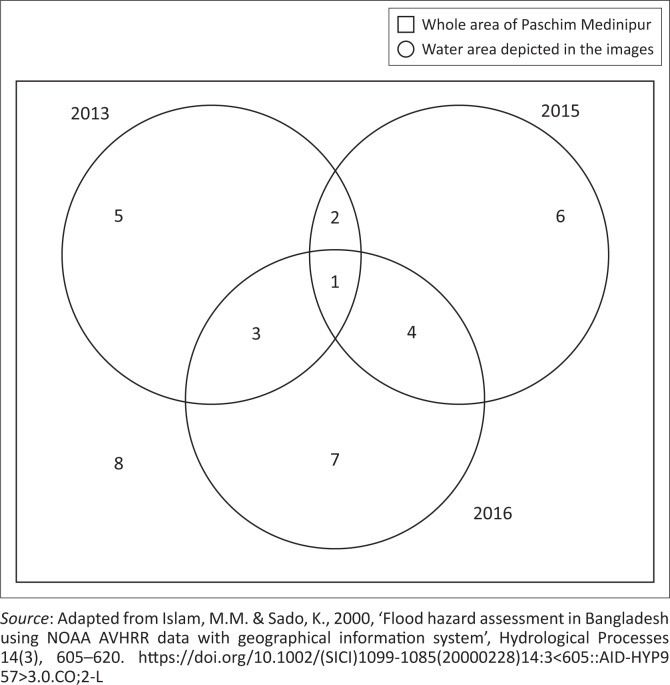
Computational protocol of flood affected frequency.

The notion of the frequency of flood has been adopted from Islam and Sado (2000). The water and non-water areas have been classified from 9 year images and superimposed for generating a map that can record the frequency of flood (see Table 2). Firstly, the classified image of 2007, 2008 and 2009 has been associated to formulate an individual flood frequency map. The method is followed throughout the year. By this method, a three-flood compass map has been prepared. And this three-flood frequency map is assembled to formulate a flood-affected frequency map (see Figure 5).

**TABLE 2:** Classification of flood-affected frequency.

Serial Number	Map	Map	Map	Area in km^2^	Percentage of the area (%)	Assignment of class
1	W	W	W	1063.94	11.36	4
2	NW	W	W	265.35	2.83	3
3	W	NW	W	115.68	1.26	3
4	NW	W	W	185.25	1.98	3
5	W	NW	NW	53.16	0.57	2
6	NW	W	NW	112.73	1.20	2
7	NW	NW	W	68.49	0.73	2
8	NW	NW	NW	7499.00	80.09	1

Map 1 comprises classified maps of 2007, 2008 and 2009; Map 2 represents 2011, 2012 and 2013; Map 3 represents 2014, 2015 and 2016.

W, Water, NW, non-water.

**FIGURE 6:**
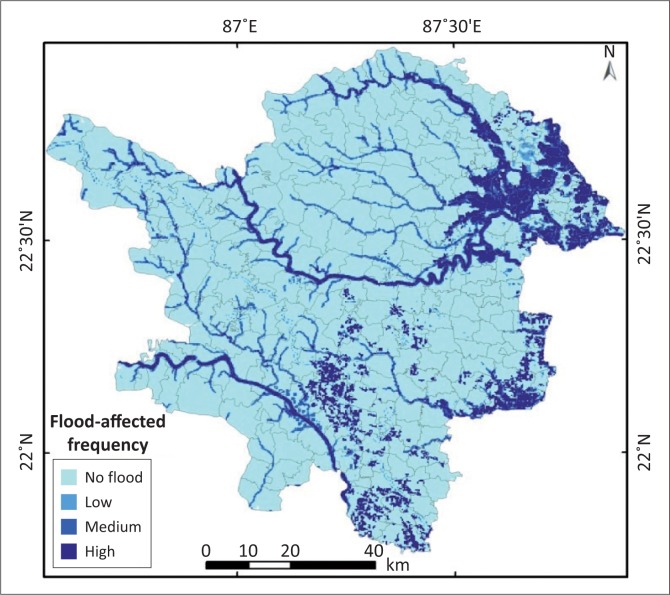
Flood affected frequency map.

When the inundated area is found in all three images, then it is identified as the worst affected area and hence it is the most vulnerable zone. The inundated identical area when found in two out of three images is identified as the medium to low affected area. If in any of the images inundated areas are not found, then this is classified as a non-flooded area (Figure 6). The final map of flood-affected frequency has been reclassified again into four types as per the intensity of inundation, assembling to flood rankings order class 1, class 2, class 3 and class 4 as no-flood, low flood, medium flood and high flood hazards areas, respectively.

The flood-affected frequency map revealed that around 12% of the study area in the worst affected area is prone to regular inundation. Around 80% of the study area falls under non-flood category and around 8% of the area has been subjected to medium to low flood categories.

### Flood water depth analysis

**FIGURE 7:**
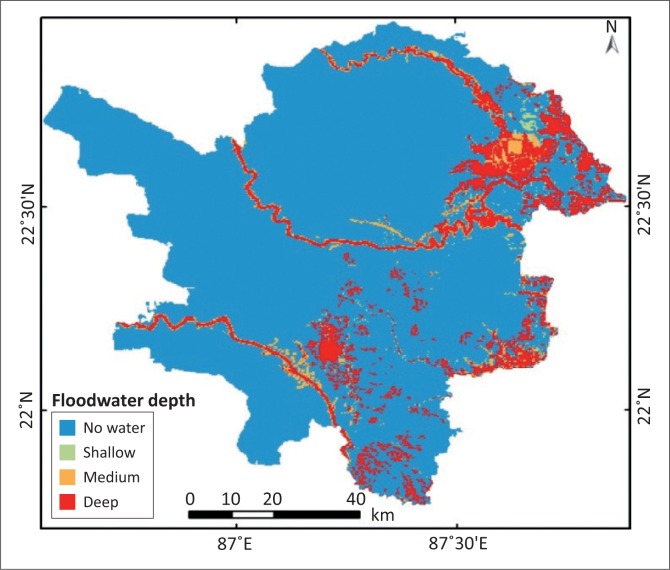
Flood water depth map.

For developing the series of flood water depth maps, DEM and data of the main rivers have been used. The flood depth maps have been reclassified into four categories: no water, shallow water, medium water and deep water. The model builder utility of Arc GIS has been used to bring out the flood water depth map for the study area. If in the same image deep flood and a medium flood are noticed at least once in the other images, then it has been identified as deep water; if in two images it is found medium, then it has been considered medium water; if in two images it is found shallow, then it has been considered shallow water. The remaining areas are considered as non-flooded areas (Figure 7).

Flood depth maps revealed that around 1025 km^2^ area occupied deep flood depth categories and medium, shallow and non-flood area occupied 2.26%, 1.21%, 85%, respectively (see Table 4). Here, the depth maps developed only the basis of topographical conditions during the formation of the elevation model of the study area and field visit (see Table 3).

**TABLE 3:** Flood water depth with type.

Type of depth	Depth in metre
Deep	> 3.50
Medium	2–3.50
Shallow	< 2

**TABLE 4:** Type of floodwater depth and occupied area.

Type of depth	Area in km^2^	Percentage of the area
No water	8014.25	85.59
Shallow	113.07	1.21
Medium	211.77	2.26
Deep	1024.60	10.94

### Flood hazard map preparation

Geospatial techniques, particularly Remote Sensing (RS), are the most reliable techniques for the assessment of flood hazard (McKean, Buechel & Gaydo 1991). The final hazard map has been derived from combining the flood-affected frequency maps and flood depth maps. If a cell marks non-flooded in both maps, then it has been regarded as a non-hazard zone. If a cell represents low flooded areas in flood-affected frequency map and shallow flood water depth in flood water depth maps, then it is regarded as a low hazard zone. If a cell represents medium flooded areas in flood-affected frequency map and medium flood water depth in flood water depth maps, then it is regarded as a medium hazard zone. And then if a cell represents deep flood water in flood water depth maps and high flood areas in flood-affected frequency map, then it is regarded as a high hazard zone.

**FIGURE 8:**
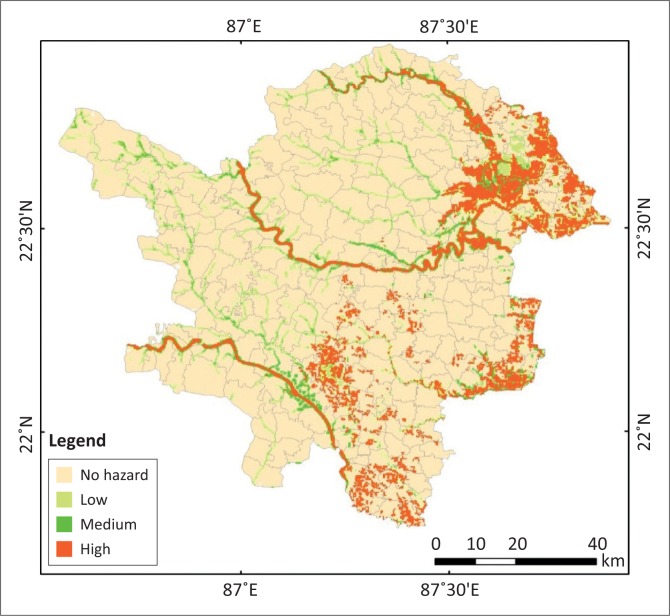
Flood hazard map obtained from flood-affected frequency and flood water depth together.

**FIGURE 9:**
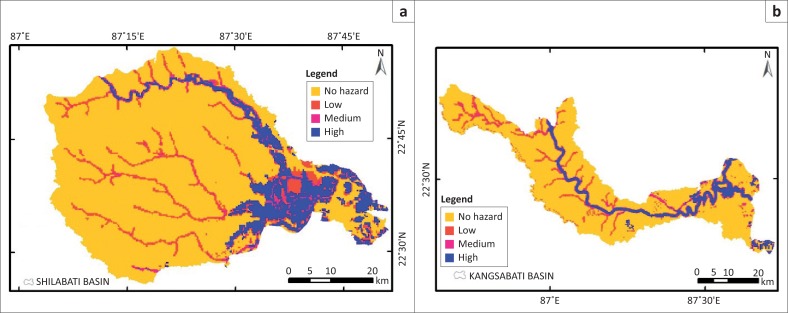
(a) Flood hazard map of Silabati Catchment and (b) Kangsabati Catchment.

**FIGURE 10:**
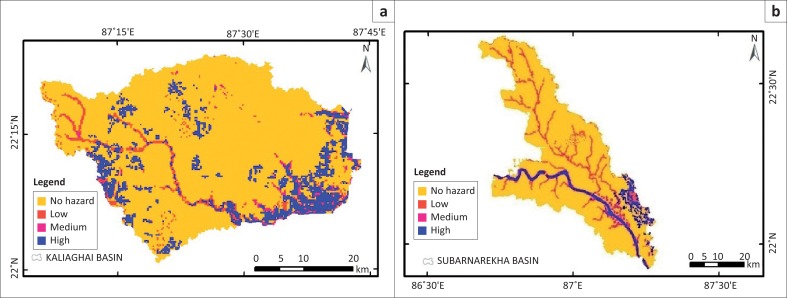
(a) Flood hazard map of Kaliaghai Catchment and (b) Subarnarekha Catchment.

On the basis of hazard intensity, again the flood hazard map has been reclassified into four types: class 1, class 2, class 3 and class 4 as no hazard, low hazard, medium hazard and high hazard zone, respectively (see Figures 8,9,10). The final flood hazard map was incorporated with population and housing data of 2011 census to assess the vulnerability of these two components to flood hazard. Table 5 presents the distribution of population and Table 6 reveals the distribution of housing units in different flood hazard zones. In Silabati river catchment, high flood hazard area is maximum, approximately 313.60 km^2^, and in Subarnarekha river catchment it is minimum at approximately 118.50 km^2^ (see Table 7).

**TABLE 5:** Vulnerability of the population according to flood hazards zone.

Hazard zone	Population (%)	Female (%)	Children (%)
High	24.00	21.00	23.00
Medium	6.35	11.00	9.53
Low	22.13	24.74	22.75
No hazard	47.52	43.53	44.72

**TABLE 6:** Vulnerability of human settlement according to flood hazards zone.

Hazard zone	Katcha houses (%)	Semi-pucca houses (%)	Pucca houses (%)
High	16.51	13.54	8.19
Medium	11.15	8.48	7.52
Low	23.63	20.36	18.00
No hazard	48.71	57.62	66.29

**TABLE 7:** Catchment-wise flood affected area.

Name of the river basin	Hazard zone	Flood affected area (km^2^)
Kaliaghai	No Hazard	1375.71506966000
	Low	69.67509318830
	Medium	24.98806735580
	High	203.52481868300
	Total	1673.90304888710
Kangsabati	No Hazard	1332.58596256000
	Low	117.73146585800
	Medium	68.81252244250
	High	148.75791790400
	Total	1667.88786876450
Subarnarekha	No Hazard	1512.78406028000
	Low	149.53886102400
	Medium	69.46175673590
	High	118.50048219100
	Total	1850.28516023090
Silabati	No Hazard	2153.17958182000
	Low	220.28478782300
	Medium	65.44897202360
	High	313.59554883200
	Total	2752.50889049860

## Conclusion

The vulnerability analysis exposed that 24% of the total population in the study area has been exposed to high flood hazard zone and 6.35%, 22.13%, 47.52% of population has been exposed to medium, low and no hazards zones, respectively. Similarly, 21% female population and 23% child population have been exposed to high flood hazard zones. Around 35.74% female population and 32.28% child population have been exposed to low to medium hazard zones. Almost 43.53% female population and 44.72% child population have been exposed to no hazard zones. In addition to the housing types, almost 16.51% katcha, 15.54% semi-pucca and 10.19% pucca houses have been exposed to high hazard zones. Around 34.78% katcha, 26.84% semi-pucca and 25.52% pucca houses are exposed to medium to low hazard zones. The analysis of the study clearly indicates that floods remain a significant threat to the people in Paschim Medinipur. As observed in Paschim Medinipur district, people set up their settlements on the floodplains and many brick-kiln industries are also set up on the floodplains which destroy the stiffness of the river embankment. Therefore, the flood hazards increased rapidly. That is why to ameliorate the flood-induced damage, the derived flood hazard map is worthless. Despite the above work, a study should be undertaken to assess the vulnerability and risk of flooding on physical and demographical variables. Therefore, research on flood effects may provide a deeper understanding of flooding in the study area or elsewhere.

In developing countries, where exact information is seriously lacking, such hazard maps are extremely useful for saving the lives and property of millions of people, particularly marginal groups.

## References

[CIT0001] Bates P.D., Wilson M.D., Horritt M.S., Mason D.C., Holden N. & Currie A., 2006, ‘Reach scale floodplain inundation dynamics observed using airborne synthetic aperture radar imagery: Data analysis and modelling’, *Journal of Hydrology* 328, 306–318. https://doi.org/10.1016/j.jhydrol.2005.12.028

[CIT0002] Dingman S.L., 1975, ‘An expedient approach to community wide flood plain delineation and regulation’, Paper Presented at Annual Meeting, AGU, Washington, DC, 17th June.

[CIT0003] Ellis D.W., 1969, ‘Flood plain mapping by the U. Sr Geological Survey’, in Dougal M.D. (ed.), *Floodplain management: Lowa’s experience*, pp. 197–206, Lowa State University Press, Ames, LA.

[CIT0004] Huang C., Chen Y. & Wu J., 2014, ‘Mapping spatio-temporal flood inundation dynamics at large river basin scale using time-series flow data and MODIS imagery’, *International Journal of Applied Earth Observation and Geoinformation* 26, 350–362. https://doi.org/10.1016/j.jag.2013.09.002

[CIT0005] Islam A.S., Bala S.K. & Haque M.A., 2010, ‘Flood inundation map of Bangladesh using MODIS time-series images’, *Journal of Flood Risk Management* 3, 210–222. https://doi.org/10.1111/j.1753-318X.2010.01074.x

[CIT0006] Islam M.M. & Sado K., 2000, ‘Flood hazard assessment in Bangladesh using NOAA AVHRR data with geographical information system’, *Hydrological Processes* 14(3), 605–620. https://doi.org/10.1002/(SICI)1099-1085(20000228)14:3<605::AID-HYP957>3.0.CO;2-L

[CIT0007] McKean J., Buechel S. & Gaydo L., 1991, ‘Remote sensing and landslide hazard assessment’, *Photogrammetric Engineering & Remote Sensing* 57(9), 1185–1193.

[CIT0008] Sharma S.V., Chakravarthi V., Srinivasarao G. & Bhanumurthy V., 2016, ‘Extraction of detailed level flood hazard zones using multi-temporal historical satellite data-sets – A case study of Kopili River Basin, Assam, India’, *Geomatics, Natural Hazards and Risk* 20, 1–11. https://doi.org/10.1080/19475705.2016.1265014

[CIT0009] Wolman M.G., 1971, ‘Evaluating alternative techniques of flood-plain mapping’, *Water Resources Research* 7(6), 1383–1392.

